# Compare the differences of synonymous codon usage between the two species within cardiovirus

**DOI:** 10.1186/1743-422X-8-325

**Published:** 2011-06-27

**Authors:** Wen-qian Liu, Jie Zhang, Yi-qiang Zhang, Jian-hua Zhou, Hao-tai Chen, Li-na Ma, Yao-zhong Ding, Yongsheng Liu

**Affiliations:** 1 State Key Laboratory of Veterinary Etiological Biology, Lanzhou Veterinary Research Institute, Chinese Academy of Agricultural Sciences, Lanzhou 730046, Gansu, PR China

## Abstract

**Background:**

Cardioviruses are positive-strand RNA viruses in the Picornaviridae family that can cause enteric infection in rodents and also been detected at lower frequencies in other mammals such as pigs and human beings. The Cardiovirus genus consists two distinct species: Encephalomyocarditis virus (EMCV) and Theilovirus (ThV). There are a lot differences between the two species. In this study, the differences of codon usage in EMCV and ThV were compared.

**Results:**

The mean ENC values of EMCV and ThV are 54.86 and 51.08 respectively, higher than 40.And there are correlations between (C+G)_12_% and (C+G)_3_% for both EMCV and ThV (r = -0.736;r = 0.986, P < 0.01, repectively). For ThV the (C+G)_12_%, (C+G)_3_%, axis *f*'_1 _and axis *f*'_2 _had a significant correlations respectively but not for EMCV. According to the RSCU values, the EMCV species seemed to prefer U, G and C ending codon, while the ThV spice seemed to like using U and A ending codon. However, in both genus AGA for Arg, AUU for Ile, UCU for Ser, and GGA for Gly were chosen preferentially. Correspondence analysis detected one major trend in the first axis (*f*'_1_) which accounted for 22.89% of the total variation, and another major trend in the second axis (*f*'_2_) which accounted for 17.64% of the total variation. And the plots of the same serotype seemed at the same region at the coordinate.

**Conclusion:**

The overall extents of codon usage bias in both EMCV and ThV are low. The mutational pressure is the main factor that determines the codon usage bias, but the (C+G) content plays a more important role in codon usage bias for ThV than for EMCV. The synonymous codon usage pattern in both EMCV and ThV genes is gene function and geography specific, but not host specific. Maybe the serotype is one factor effected the codon bias for ThV, and location has no significant effect on the variations of synonymous codon usage in these virus genes.

## Background

Synonymous codon usage is biased and the bias seems to be different in different organisms[1,2]. Many factors are concerned to be the reasons for this bias, such as degree and timing of gene expression, codon-anticodon interactions, transcription and translation rate and fidelity, codon context, and global and local (C+G) content[3,4]. Understanding the extent and causes of biases in codon usage is essential to the understanding of viral evolution, particularly the interplay between viruses and the immune response [5]. More recent studies have revealed that patterns of codon usage bias and nucleotide composition within many cellular genomes are far more complex than previously imagined, and the factors shaping their evolution are still not entirely understood. In general, natural selection and/or mutation pressure for accurate and efficient translation in various organisms are the main reasons to this bias. In addition, compared with natural selection, mutation pressure plays an important role in synonymous codon usage pattern in some RNA viruses [6-10].

*Picornaviruses *are positive single-stranded RNA viruses that cause a variety of important disease states in humans and animals, such as foot-and-mouth disease. The Cardiovirus genus of the family *Picornaviridae *consists two distinct species: *Encephalomyocarditis *virus (EMCV) and *Theilovirus *ThV [11]. The EMCVs comprise a single serotype and have a wide host range [11-21], while the ThV species, probably includes four serotypes: Theiler's murine encephalomyelitis virus (TMEV), Vilyuisk human encephalomyelitis virus (VHEV), Thera virus (TRV; isolated from rats) and Saffold virus (SAFV; isolated from humans) 1-8., which appear to have much narrower host ranges than EMCV. Like the other virus within *Picornaviruses *family, the strains in Cardiovirus also consist a open-read-frame (ORF), 5'-untranslate region (5'-UTR) and 3'-untranslate region (3'-UTR). However there are still many complete nucleotide sequences of this type are not reported especially, such as SAFV, therefore there is much more work to study this type virus.

Nevertheless, little information about codon usage pattern of Cardiovirus genus genome including the relative synonymous codon usage (RSCU) and codon usage bias (CUB) in the process of its evolution is available. In this study, the key genetic determinants of codon usage index in Cardiovirus genus were examined.

## Results

### The characteristics of Synonymous codon usage in EMCV and ThV

In order to investigate the extent of codon usage bias in Cardiovirus, all RSCU values of different codons in 39 Cardiovirus strains were calculated. As shown in Table 1, the EMCV strains seem to like using U, G and C ending codon, while the ThV species seem to like using U and A ending codons. The values of ENC (effective number of codons) (Table 2) among EMCV strains examined are very similar, which vary from 54.40 to 56.11 with a mean value of 54.86 and S.D. of 0.36, while the values of ENC among Theilovirus are a little different, which vary from 48.24 to 54.94 with a mean value of 51.08 and S.D. of 2.17. Because all the ENC values of both EMCV strains and Theilovirus strains are high (ENC > 40), codon usage bias in Cardioviru genome is a little slight. However, there is a marked variation in codon usage pattern among different Theilovirus genes (S.D. = 6.41) compared to the EMCV genes (S.D. = 0.36). The concept is further supported by the values of CG3. The (C+G)_3_% values of EMCV strains range from 46.47 to 52.11% with a mean of 48.90 and S.D. of 2.18 while these values of ThV strains range from 37.35 to 51.00, with a mean of 43.72 and S.D. of 5.77.

**Table 1:** Synonymous codon usage in Cardiovirus genome

**AA**^**a**^	Codon	^**b**^**RSCU**	Codon	^**b**^**RSCU**
	EMCV		ThV	
Ala	GCU(A)	1.21	**GCU(A)**	**1.64**
	**GCC(A)**	**1.45**	GCC(A)	1.05
	GCA(A)	0.80	GCA(A)	1.02
	GCG(A)	0.54	GCG(A)	0.29
Arg	CGU(R)	0.91	CGU(R)	0.82
	CGC(R)	0.74	CGC(R)	0.94
	CGA(R)	0.28	CGA(R)	0.50
	CGG(R)	0.61	CGG(R)	0.32
	**AGA(R)**	**2.60**	**AGA(R)**	**2.67**
	AGG(R)	0.87	AGG(R)	0.75
Asn	**AAU(N)**	**1.17**	**AAU(N)**	**1.07**
	AAC(N)	0.83	AAC(N)	0.93
Asp	**GAU(D)**	**1.17**	GAU(D)	0.98
	GAC(D)	0.83	GAC(D)	1.02
Cys	UGU(C)	0.97	**UGU(C)**	**1.23**
	UGC(C)	1.04	UGC(C)	0.77
Gln	CAA(Q)	0.83	**CAA(Q)**	**1.26**
	**CAG(Q)**	**1.17**	CAG(Q)	0.74
Glu	GAA(E)	0.93	**GAA(E)**	**1.38**
	GAG(E)	1.07	GAG(E)	0.62
Gly	GGU(G)	1.06	GGU(G)	1.14
	GGC(G)	0.92	GGC(G)	1.01
	**GGA(G)**	**1.25**	**GGA(G)**	**1.53**
	GGG(G)	0.77	GGG(G)	0.32
His	**CAU(H)**	**1.23**	CAU(H)	0.84
	CAC(H)	0.77	**CAC(H)**	**1.16**
Ile	**AUU(I)**	**1.61**	**AUU(I)**	**1.55**
	AUC(I)	0.66	AUC(I)	0.85
	AUA(I)	0.73	AUA(I)	0.60
Leu	UUA(L)	0.43	UUA(L)	0.73
	UUG(L)	1.40	**UUG(L)**	**1.40**
	CUU(L)	0.95	CUU(L)	1.35
	CUC(L)	1.03	CUC(L)	1.25
	CUA(L)	0.78	CUA(L)	0.49
	**CUG(L)**	**1.41**	CUG(L)	0.77
Lys	AAA(K)	0.83	**AAA(K)**	**1.15**
	**AAG(K)**	**1.17**	AAG(K)	0.85
Phe	UUU(F)	1.10	UUU(F)	1.08
	UUC(F)	0.90	UUC(F)	0.92
Pro	CCU(P)	1.04	**CCU(P)**	**1.42**
	CCC(P)	1.12	CCC(P)	1.16
	**CCA(P)**	**1.37**	CCA(P)	1.02
	CCG(P)	0.47	CCG(P)	0.40
Ser	AGU(S)	0.60	AGU(S)	0.53
	AGC(S)	0.52	AGC(S)	0.45
	**UCU(S)**	**1.57**	**UCU(S)**	**1.82**
	UCC(S)	1.36	UCC(S)	1.44
	UCA(S)	1.40	UCA(S)	1.37
	UCG(S)	0.55	UCG(S)	0.39
Thr	ACU(T)	1.27	**ACU(T)**	**1.49**
	**ACC(T)**	**1.31**	ACC(T)	1.21
	ACA(T)	1.13	ACA(T)	1.05
	ACG(T)	0.29	ACG(T)	0.25
Tyr	UAU(Y)	0.99	UAU(Y)	0.94
	UAC(Y)	1.01	UAC(Y)	1.06
Val	GUU(V)	0.90	**GUU(V)**	**1.53**
	GUC(V)	0.96	GUC(V)	0.94
	GUA(V)	0.66	GUA(V)	0.62
	**GUG(V)**	**1.48**	GUG(V)	0.91

a Amino acid

b The RSCU value is a mean value of each codon for a particular

amino acid

**Table 2:** Identified nucleotide contents in complete coding region (length > 250 bps) in the Cardiovirus genome

**NO**.	T(U)	C	A	G	T-3	C-3	A-3	G-3	(C+G)_3_%	**(C+G)**_**12**_**%**	ENC
	
EMCV											
1	25.37	24.67	26.30	23.67	28.13	25.38	22.02	24.47	49.85	47.58	54.70
2	25.37	24.67	26.30	23.67	28.09	25.43	21.94	24.55	49.98	47.51	54.75
3	25.44	24.64	26.22	23.70	28.17	25.38	21.98	24.47	49.85	47.58	54.67
4	25.35	24.71	26.28	23.65	28.04	25.47	21.98	24.51	49.98	47.56	54.73
5	25.37	24.71	26.31	23.61	19.89	20.72	27.56	31.84	52.55	46.21	56.11
6	25.29	24.77	26.22	23.71	28.00	25.56	21.89	24.55	50.11	47.67	54.88
7	25.43	24.64	26.24	23.70	28.22	25.34	21.89	24.55	49.89	47.56	54.88
8	25.37	24.70	26.28	23.65	28.13	25.38	21.98	24.51	49.89	47.58	54.80
9	25.35	24.80	26.29	23.56	28.04	28.09	29.31	14.57	42.65	51.21	54.88
10	25.32	24.76	26.25	23.67	28.09	25.43	21.98	24.51	49.93	47.67	54.84
11	26.52	23.76	26.32	23.41	31.39	22.76	22.10	23.76	46.51	47.49	55.10
12	25.40	24.68	26.28	23.64	28.17	25.38	21.98	24.47	49.85	47.56	54.58
13	25.89	23.84	26.78	23.49	29.22	23.51	23.20	24.07	47.58	47.21	54.59
14	25.34	24.61	26.49	23.56	28.09	25.56	22.07	24.29	49.85	47.34	55.06
15	25.92	23.81	26.75	23.52	29.22	23.46	23.24	24.07	47.54	47.23	54.40
16	25.86	23.83	26.76	23.55	29.18	23.55	23.16	24.12	47.67	47.23	54.64
17	25.38	24.68	26.25	23.68	28.09	25.38	21.94	24.60	49.98	47.56	54.93
18	26.55	23.74	26.30	23.41	31.43	22.76	22.10	23.71	46.47	47.49	55.01
ThV											

1	26.95	27.33	24.31	21.41	32.51	32.99	16.93	17.58	50.56	47.83	52.40
2	26.92	27.33	24.31	21.44	32.47	32.94	16.93	17.66	50.61	47.85	52.50
3	27.37	27.09	24.17	21.37	32.97	33.10	16.77	17.16	50.26	47.57	52.48
4	26.84	27.52	24.45	21.20	32.42	33.42	17.23	16.93	50.35	47.89	52.29
5	27.03	27.56	24.23	21.18	32.51	33.90	16.75	16.84	50.74	47.74	51.90
6	26.90	27.52	24.34	21.24	31.99	33.85	17.01	17.14	51.00	47.64	52.48
7	27.21	26.97	24.13	21.69	33.81	31.29	16.58	18.32	49.61	48.18	52.58
8	25.55	25.69	27.44	21.32	28.64	29.42	23.66	18.28	47.70	46.66	54.61
9	25.64	25.58	27.37	21.42	28.90	28.90	23.57	18.63	47.53	46.73	54.68
10	25.61	25.53	27.54	21.32	29.07	28.55	24.22	18.15	46.71	46.92	54.94
11	28.33	22.36	29.53	19.78	34.00	21.68	28.65	15.67	37.35	44.54	48.62
12	27.87	22.77	29.38	19.98	33.44	22.67	27.11	16.78	39.45	44.40	50.34
13	27.71	23.05	29.95	19.28	32.62	23.69	28.05	15.64	39.33	43.84	49.83
14	28.05	22.63	29.89	19.43	33.89	22.43	27.79	15.90	38.33	43.92	49.48
15	27.93	22.79	29.89	19.38	33.45	22.91	27.96	15.68	38.59	43.97	49.26
16	27.95	22.65	29.67	19.73	33.28	22.43	27.70	16.59	39.02	44.05	50.13
17	27.73	23.11	30.08	19.08	32.75	23.69	28.40	15.16	38.85	43.86	49.67
18	28.09	22.52	29.98	19.41	33.75	22.34	28.27	15.64	37.98	43.90	48.47
19	28.15	22.40	30.02	19.43	33.89	22.13	28.18	15.81	37.94	43.77	48.47
20	28.19	22.42	30.04	19.35	33.80	22.30	28.31	15.59	37.89	43.71	48.24
21	27.82	22.94	30.11	19.13	33.10	23.21	28.48	15.20	38.42	43.90	49.35

### Compositional properties of coding sequences of both EMCV and ThV

**Figure 1:**
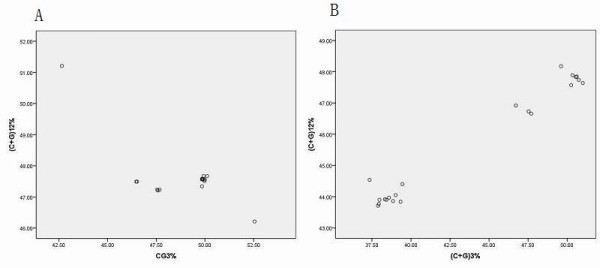
**Correlation between (C+G) content at first and second codon positions (C+G)_12 _with that at synonymous third codon positions (C+G)_3_**. *A for EMCV. B for ThV.

**Figure 2:**
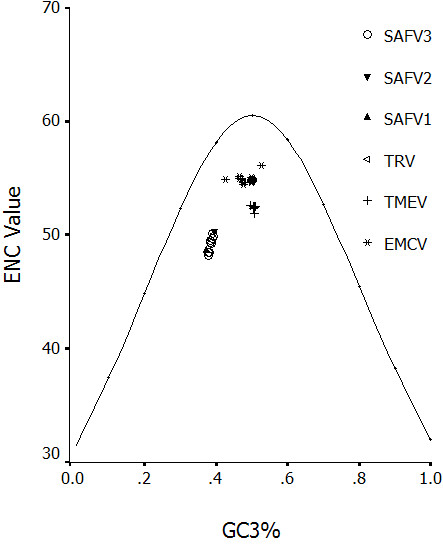
**Effective number of codons used in each ORF plotted against the (C+G)_3_**. The continuous curve plots the relationship between (C+G)_3_. and ENC in the absence of selection. All of spots lie below the expected curve.

As shown in Table 3, (C+G)% has a highly significant correlation with each A_3_%, C_3_%, G_3_% and U_3_% . (C+G)_3_% has a highly significant correlation with each of A%, U%, C% and G% among the ThV strains but not among the EMCV strains. This indicates that the (C+G)% and (C+G)_3_% may reflect some more important characteristics of codon usage pattern of ThV compared with EMCV. Then the C+G content at first and second codon positions ((C+G)12%) was compared with that at synonymous third codon positions ((C+G)_3_%) for both EMCV and ThV respectively. A highly significant correlation is observed in ThV (r = 0.986, P < 0.01)(Figure 1A, Table 4). However for EMCV a highly negative correlation is observed (r = -0.736, P < .0.01)(Figure 1B, Table 4). Then the (C+G)_12_% and (C+G)_3_% of both EMCV and ThV were compared with axis *f*'_1 _and axis *f*'_2 _respectively. The results (Table 4) show that for EMCV there are no significant correlations between (C+G)_3_%, (C+G)_12_%, axis *f*'_1 _and axis *f*'_2 _are observed, while the results of ThV are opposite. The ENC-plot [ENC plotted against (G+C)_3_%] was used as a part of general strategy to investigate patterns of synonymous codon usage and all of the spots lie below the expected curve (Figure 2). All these results imply that the codon bias of Cardiovirus especially the ThV can be explained mainly by an uneven base composition, in other words, by mutation pressure rather than natural selection and the (C+G) content has a more significant effect for ThV than EMCV

**Table 3:** Summary of correlation analysis between the A, U, C, G contents and A_3_, U_3_, C_3_, G_3 _contents in all selected samples.

EMCV		U3%	C3%	A3%	G3%	**(C+G)**_**3**_**%**
	U%	r = 0.571*	r = -0.585*	r = -0.109	r = -0.053	r = -0.509*
	C%	r = -0.533*	r = 0.605**	r = 0.108	r = 0.013	r = 0.468
	A%	r = 0.184	r = -0.355	r = 0.086	r = -0.01	r = -0.279
	G%	r = -0.42	r = 0.508*	r = -0.191	r = 0.185	r = 0.634**
	CG%	r = -0.524*	r = 0.601**	r = 0.054	r = 0.046	r = 0.511*
ThV						

	U%	r = 0.941**	r = -0.654**	r = 0.504*	r = -0.848 **	r = -0.722 **
	C%	r = -0.410	r = 0.998**	r = -0.975 **	r = 0.741**	r = 0.994**
	A%	r = 0.245	r = -0.976**	r = 0.997**	r = -0.699**	r = -0.967 **
	G%	r = -0.549**	r = 0.910**	r = -0.887**	r = 0.923**	r = 0.955 **
	CG%	r = -0.461 *	r = 0.986 **	r = -0.963 **	r = 0.811**	r = 0.998 **

* means 0.01 < p < 0.05

**means p < 0.01

**Table 4:** Analysis of correlation between the first two principle axes and nucleotide contents in samples.

EMCV		**(C+G)**_**3**_**%**	**(C+G)**_**12**_**%**	*f_***1***_'*	*f_***2***_'*
	(C+G)_3_%	r = 1	r = -0.736**	r = 0.360	r = 0.357
	(C+G)12%	r = -0.736**	r = 1	r = 0.202	r = 0.139
	*f_1_'*	r = 0.360	r = 0.202	r = 1	r = 0.612**
	*f_2_'*	r = 0.357	4 = 0.139	r = 0.612**	r = 1
ThV					

	(C+G)_3_%	r = 1	r = 0.986**	r = 0.979**	r = 0.973**
	(C+G)_12_%	r = 0.986**	r = 1	r = 0.971**	r = 0.969**
	*f_1_'*	r = 0.973**	r = 0.971**	r = 1	r = 0.917**
	*f_2_'*	r = 0.979**	r = 0.969**	r = 0.917**	r = 1

* means 0.01 < p < 0.05

**means p < 0.01^©^

### Correspondence analysis (COA) for all the strains

**Figure 3:**
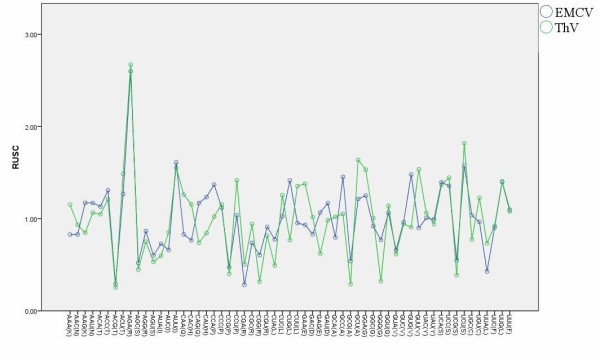
**Compare the codon the codon usage pattern among EMCV and Thv**.

To investigate the major trend in codon usage variation among Cardiovirus, COA was used for all 39 Cardiovirus complete coding regions selected for this study. COA detect one major trend in the first axis (*f*'_1_) which account for 22.89% of the total variation, and another major trend in the second axis (*f*'_2_) which account for 17.64% of the total variation. The coordinate of the complete coding region of each gene was plotted in Figure 3 defining by the first and second principal axes. It is clear that the *f*'_1 _values of all EMCV are positive while the ThVs are negative. And the plots of the strains of the same serotype seem at the same region. Furthermore, the EMCV has a tendency to converge tightly while the different serotypes of ThV are dispersed. These findings imply that different serotype may have different codon usage patterns. Interestingly, the plot of EMC-30 is a little far from the other EMCV, but this does not indicate the location is an element that could dramatically influence the codon usage pattern.

### Qualitative evaluation of codon usage bias in EMCV and ThV

**Figure 4:**
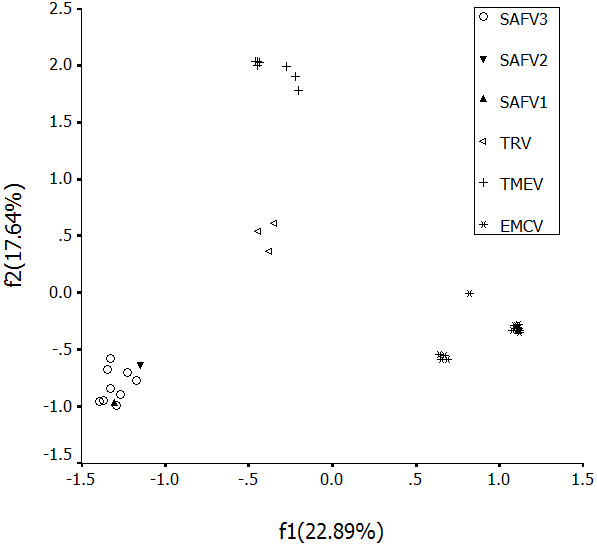
**A plot of value of the first and second axis of each complete coding region in COA**. The first axis (*f*'_1_) accounts for22.89% of the total variation, and the second axis (*f*'_2_) accounts for 17.64% of the total variation.

There was a seemingly random variation in RSCU between amino acids and gene groups. There were several synonymous codons with strong discrepancy for codon usage in each genus. As for EMCV, in details, AGA for Arg, GGA for Gly, CAU for His, AUU for Ile, CCA for Pro, UCU for Ser and GUG for Val. And there are some differences of the global pattern of codon usage between EMCV and Theilovirus. However, in both genus AGA for Arg, AUU for Ile, UCU for Ser, and GGA for Gly were chosen preferentially (Figure 4).

## Discussion

Studies of synonymous codon usage in viruses can reveal much about viral genomes. In this study, we used RSCU, ENC, COA, and GC_3S_, to measure the synonymous codon usage bias in order to compare the differences between EMCV and ThV, the two species within Cardiovirus. The synonymous codon usage bias in coding regions of both EMCV and ThV are low because the mean ENC values of 54.86 and 51.08 respectively (higher than 40). This is in agreement with previous reports about some other RNA viruses, for example, BVDV (mean ENC = 51.42), H5N1 (mean ENC = 50.91) and SARS-covs (mean ENC = 48.99)[6,7,21]. A low codon usage bias is advantageous to replicate efficiently in vertebrate host cells, with potentially distinct codon preferences. However there is a marked variation in codon usage pattern among different Theilovirus genes (S.D. = 6.41) compared to the EMCV genes (S.D. = 0.36). One explanation about this phenomenon is that the ThV probably has four serotypes while the EMCV just has one and the serotype might affect the codon choice.

A general mutational pressure, which affects the whole genome, would certainly account for the majority of the codon usage variation. In this study, the general association between codon usage bias and base composition suggests that mutational pressure, rather than natural selection, is mainly supported by the highly significant correlation between (C+G)_12_% and (C+G)_3_% (r = -0.736 for EMCV; r = 0.986 for ThV, P < 0.01), since the effects are present at all codon positions. Also the (G+C) content was another factor which was found to be strong correlated with codon usage bias. In this study, the results indicated the (C+G) content played an important role in codon usage bias for ThV (Table 3), but not for EMCV. This is a little complex for EMCV and we need to do more research for this genus such as each nucleotide composition, gene structure and so on to find the main factor for codon bias of EMCV. Nevertheless we still consider that the mutational pressure rather than natural selection is the one of the main factors responsible for the variation of synonymous codon usage among ORF coding sequences in Cardiovirus genus.

Generally, previous reports indicates that many viruses including foot-and-mouth disease viruses, influenza A virus subtype H5N1, severe acute respiratory syndrome Coronavirus (SARSCoV) and human bocavirus, preferentially use C and G-ended codons[2,7,9,10]. In this study we found that the EMCV strains seemed to like using U, G and C ending codon, while the ThV species seemed to like using U and A ending codon. Also there was a seemingly random variation in RSCU between amino acids and gene groups. This may be because using these codon with different endings could be advantage for replicating efficiently in host cells with potentially distinct codon preferences for both EMCV and ThV.

Serotype may be one factor for codon bias in Cardiovirus as the Figure 3 showed. And there was no evidence supported that location could be a factor for codon bias, because the plot of EMC-30 which was isolated from USA was a little far from other EMCV that were isolated from USA plots.

## Conclusion

The overall extents of codon usage bias in both EMCV and ThV are low (mean ENC = 54.86; mean ENC = 51.08 respectively, higher than 40). The mutational pressure rather than natural selection is the main factor that determines the codon usage bias that is supported by the highly significant correlation between (C+G)_12_% and (C+G)_3_% (r = -0.736 for EMCV; r = 0.986 for ThV, P < 0.01), but the (C+G) content plays a more important role in codon usage bias for ThV than for EMCV. The synonymous codon usage pattern in both EMCV and ThV genes is gene function and geography specific, but not host specific. Maybe the serotype is one factor effected the codon bias for ThV, and location has no significant effect on the variations of synonymous codon usage in these virus genes.

## Materials and methods

### Sequences

A total of 39 Cardiovirus genomes were used in this study, including 18 EMCV genomes and 21ThV genomes. The CDS of these viruses were obtained from NCBI http://www.ncbi.nlm.nih.gov/Genbank/ randomly in December 2010. And the serial number (SN), GenBank number, genotype and other detail information are listed in Table 5.

**Table 5:** The information of the 39 sequences used in this study

SN	Strain	Species	Serotype	Location	Accession No.
1	BEL-2887A/91	EMCV	EMCV	Belgium	AF356822
2	BJC3	EMCV	EMCV	China	DQ464062
3	HB1	EMCV	EMCV	China	DQ464063
4	GX0601	EMCV	EMCV	China	FJ604852
5	GX0602	EMCV	EMCV	China	FJ604853
6	GXLC	EMCV	EMCV	China	FJ897755
7	NJ08	EMCV	EMCV	China	HM641897
8	EMCV-CBNU	EMCV	EMCV	South Korea	DQ517424
9	K3	EMCV	EMCV	South Korea	EU780148
10	K11	EMCV	EMCV	South Korea	EU780149
11	M	EMCV	EMCV	Uganda	L22089
12	pEC9	EMCV	EMCV	USA	DQ288856
13	PV2	EMCV	EMCV	Panama	X87335
14	EMC-30	EMCV	EMCV	USA	AY296731
15	EMCV-D	EMCV	EMCV	Panama	M37588
16	EMCV-B	EMCV	EMCV	Panama	M22457
17	PV21	EMCV	EMCV	Panama	X74312
18	Rz-pMwt	EMCV	EMCV	USA	DQ294633
19	DG VII(1)	ThV	TMEV	USA	X56019
20	DG VII(2)	ThV	TMEV	USA	M20562
21	DA	ThV	TMEV	USA	M20301
22	BeAn 8386	ThV	TMEV	Brazil	M16020
23	TO4(B15)	ThV	TMEV	USA	EU718732
24	TO Yale	ThV	TMEV	USA	EU723238
25	Vie415HTR	ThV	TMEV	USA	EU718733
26	NGS910	ThV	TRV		AB090161
27	Rat theilovirus	ThV	TRV	USA	EU815052
28	Rat theilovirus 2008	ThV	TRV	USA	EU542581
29	California/81	ThV	SAFV-1	USA	EF165067
30	UC1	ThV	SAFV-2		EU376394
31	Nijmegen2007	ThV	SAFV-3	Netherlands	FM207487
32	D/VI2273/2004	ThV	SAFV-3	Germany	DQ294633
33	D/VI2223/2004	ThV	SAFV-3	Germany	EU681179
34	NL1999-590	ThV	SAFV-3	Netherlands	HM181996
35	BCH115	ThV	SAFV-3	China	GU943514
36	NL2007-2686	ThV	SAFV-3	Netherlands	HM181997
37	NL2007-2690	ThV	SAFV-3	Netherlands	HM181998
38	NL2005-1035	ThV	SAFV-3	Netherlands	HM181999
39	BCH1031	ThV	SAFV-3	China	GU943513

### Measures of relative synonymous codon usage

Relative synonymous codon usage (RSCU) values of each codon in each ORF were used to measure the synonymous codon usage. RSCU values are largely independent of amino acid composition and are particularly useful in comparing codon usage between genes, or sets of genes that differ in their size and amino acid composition [22]. The RSCU value of the *i*th codon for the *j*th amino acid was calculated as:

Where g_ij _is the observed number of the *i*th codon for *j*th amino acid which has n_i _type of synonymous codons. When the codon with RSCU values close to 1.0, it means that this codon is chosen equally and randomly. The values of RSCU were obtained by CodonW program

The effective number of codons (ENC) was calculated to quantify the codon usage bias of an ORF [23], which is the best estimator of absolute synonymous codon usage bias [24]. The larger extent of codon preference in a gene, the smaller the ENC value is. In an extremely biased gene where only one codon is used for each amino acid, this value would be 20; if all codons are used equally, it would be 61; and if the value of the ENC is greater than 40, the codon usage bias was regarded as a low bias [25] The values of ENC were obtained by CodonW program.

Composition analysis of coding region

In order to better understand the synonymous codon usage variation among different Cardiovirus isolates, The (C+G) content at the first and second codon positions [(C+G)_12_%] and that at the synonymous third position [(C+G)_3_%] were calculated by the CodonW program, respectively [26,27]. The values of the (C+G) content at different positions were used to compare with the values of the other compositional content.

### Correspondence analysis (COA)

Multivariate statistical analysis can be used to explore the relationships between variables and samples. In this study, correspondence analysis was used to investigate the major trend in codon usage variation among genes. In this study, the complete coding region of each gene was represented as a 59 dimensional vector, and each dimension corresponds to the RSCU value of one sense codon (excluding Met, Trp, and the termination codons) [28].

### Correlation analysis

Correlation analysis was used to identify the relationship between nucleotide composition and synonymous codon usage pattern [29]. This analysis was implemented based on the Spearman's rank correlation analysis way.

All statistical processes were carried out by with statistical software SPSS 11.5 for windows.

## Competing interests

The authors declare that they have no competing interests.

## Authors' contributions

WQL and JZ conceived of the study. WQL downloaded these sequences, calculated the data, analyzed the results and drafted the manuscript; JZ supervised the research, analyzed the results and helped draft the manuscript; JHZ calculated and visualized the data; YQZ, HTC, LNM and YZD assisted with data analysis; YSL supervised the research and helped draft the manuscript.
